# Encoding of facial features by single neurons in the human amygdala and hippocampus

**DOI:** 10.1038/s42003-021-02917-1

**Published:** 2021-12-14

**Authors:** Runnan Cao, Xin Li, Nicholas J. Brandmeir, Shuo Wang

**Affiliations:** 1 Lane Department of Computer Science and Electrical Engineering, West Virginia University, Morgantown, WV 26506 USA; 2 Department of Neurosurgery, West Virginia University, Morgantown, WV 26506 USA; 3 Department of Radiology, Washington University in St. Louis, St. Louis, MO 63110 USA

**Keywords:** Perception, Neural circuits, Human behaviour

## Abstract

Faces are salient social stimuli that attract a stereotypical pattern of eye movement. The human amygdala and hippocampus are involved in various aspects of face processing; however, it remains unclear how they encode the content of fixations when viewing faces. To answer this question, we employed single-neuron recordings with simultaneous eye tracking when participants viewed natural face stimuli. We found a class of neurons in the human amygdala and hippocampus that encoded salient facial features such as the eyes and mouth. With a control experiment using non-face stimuli, we further showed that feature selectivity was specific to faces. We also found another population of neurons that differentiated saccades to the eyes vs. the mouth. Population decoding confirmed our results and further revealed the temporal dynamics of face feature coding. Interestingly, we found that the amygdala and hippocampus played different roles in encoding facial features. Lastly, we revealed two functional roles of feature-selective neurons: 1) they encoded the salient region for face recognition, and 2) they were related to perceived social trait judgments. Together, our results link eye movement with neural face processing and provide important mechanistic insights for human face perception.

## Abstract (web summary)

To study how the amygdala and hippocampus encode the content of fixations when viewing faces, Cao et al. employ single-neuron recordings with simultaneous eye tracking when human participants viewed natural face stimuli. They demonstrate a link between eye movement and neural face processing to provide mechanistic insight for human face perception.

## Introduction

Primates have a dedicated visual system to process faces^1,2^. Earlier work in monkeys have revealed face-selective neurons in the inferotemporal (IT) cortex (see^3^ for a historical overview), which has been later verified in human intracranial recordings^4^. In particular, while classically investigated for their roles in long-term memory^5^, the human amygdala and hippocampus have been associated with various roles in face perception^6–9^. Lesion studies have shown that damage of the human amygdala impairs spontaneous fixations on the eyes^10^, while damage of the human hippocampus impairs face memory^11,12^. Functional data further suggested that single neurons in the human amygdala and hippocampus are not only visually selective to faces^13,14^, but also they encode specific face identities^15–17^, which forms the basis for face recognition^17,18^. Furthermore, human amygdala neurons encode facial emotions^13^ and subjective judgments of facial emotions^19^. Using a unique combination of single-neuron recordings, functional magnetic resonance imaging (fMRI), and patients with focal amygdala lesions, we have shown that the human amygdala parametrically encodes the intensity of specific facial emotions and their categorical ambiguity^20^. More broadly, single neurons in the human amygdala and hippocampus encode attention to faces^21^, as well as the memory of faces^22^. Findings from human studies are further complemented by monkey studies: single neurons in the monkey amygdala are selective not only to faces^23,24^, but also to face identities and facial expressions^25,26^.

Many aspects of face processing can be attributed to the saliency of facial features, such as the eyes and mouth^27^. However, the mechanisms by which the human amygdala and hippocampus process facial feature information and direct eye movements to salient facial features remain unclear. Neuroimaging studies have shown that amygdala activation predicts gaze direction^28^ and single neurons in the human amygdala have been found to encode whole faces compared to piecemeal faces^29^, indicating that amygdala neurons may encode facial parts, as well as holistic facial features. Using piecemeal bubbled faces, we previously demonstrated that single neurons in the human amygdala have abnormal facial feature selectivity in autism^30^. Furthermore, neurons in the monkey amygdala have been shown to encode not only the eyes but also the gaze direction when viewing a monkey face, as well as eye contact with the viewed monkey^26,31^.

In this study, we used natural face stimuli to study the neural correlates of human eye movements when participants viewed faces. We identified a subset of neurons in the human amygdala and hippocampus that differentiated visual fixations on different facial features and characterized the responses of these neurons in detail. Interestingly, we found that these facial feature-selective neurons also encoded the saliency of facial features for face identification and they were related to social trait judgments.

## Results

### Behavior

**Fig. 1:**
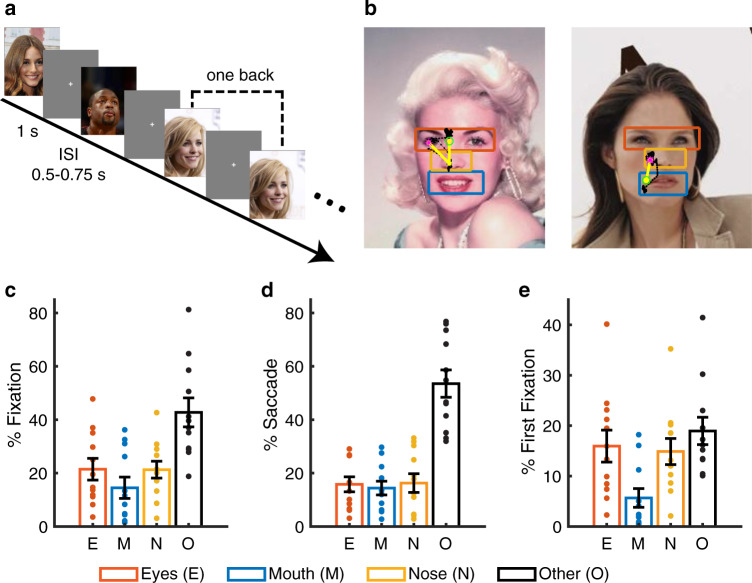
Behavior. **a** Task. We employed a one-back task in which patients responded whenever an identical famous face was repeated. Each face was presented for 1 s, followed by a jittered inter-stimulus-interval (ISI) of 0.5 to 0.75 s. Each image subtended a visual angle of approximately 10°. **b** Sample stimuli. Regions of interest (ROIs) were detected using computer vision (not shown to participants). Each yellow dot represents a fixation. Green circle: first fixation. Magenta asterisk: last fixation. Yellow line: saccades. Black dot: raw gaze position. **c** Percentage of fixation for each ROI. **d** Percentage of saccade to each ROI. **e** Percentage of first fixation onto each ROI. Error bars denote ±SEM across sessions. Each dot represents an individual session. E: eyes. M: mouth. N: nose. O: other (all other parts of the image, including hair, neck, background, etc.).

We recorded single neurons using implanted micro depth electrodes in the human amygdala and hippocampus while neurosurgical patients performed a one-back task using real-world images of famous faces (Fig. 1a; accuracy = 75.7  ±  5.28% [mean  ±  SD across sessions]). Five patients undergoing epilepsy monitoring had normal basic ability to discriminate faces and underwent 12 sessions in total (Supplementary Table 1). Participants viewed 500 natural face images of 50 celebrities (10 different photos per identity; Fig. 1b). We found that on average, 22.01 ± 13.10% of fixations were on the eyes and 13.79 ± 12.95% of fixations were on the mouth (Fig. 1b, c), consistent with previous studies^32,33^ (see also Supplementary Fig. 1 for results with normalization by region of interest [ROI] areas; note that the relatively low percentage of fixations on eyes and mouth indicated that patients might tend to avoid the eyes and mouth). Furthermore, we found that participants had 16.50 ± 9.28% of saccades to the eyes and 13.80 ± 8.49% of saccades to the mouth (Fig. 1b, d; Supplementary Fig. 1). Interestingly, among the first fixations, participants tended to fixate 10% more onto the eyes than the mouth (Fig. 1e; two-tailed paired *t* test: *t*(11) = 2.39, P = 0.036; Supplementary Fig. 1), suggesting that they sampled the eyes earlier than the mouth.

### Feature-selective neurons that discriminated fixations on the eyes vs. mouth

We isolated 422 single units across all sessions; and of these, 365 units had an overall firing rate greater than 0.15 Hz and we restricted our analysis to this subset of units, which included 178 units from the amygdala, 139 units form the anterior hippocampus, and 48 units from the posterior hippocampus (see Supplementary Table 1 for a breakdown of each individual session; see Supplementary Fig. 2 for assessment of spike sorting quality).

**Fig. 2:**
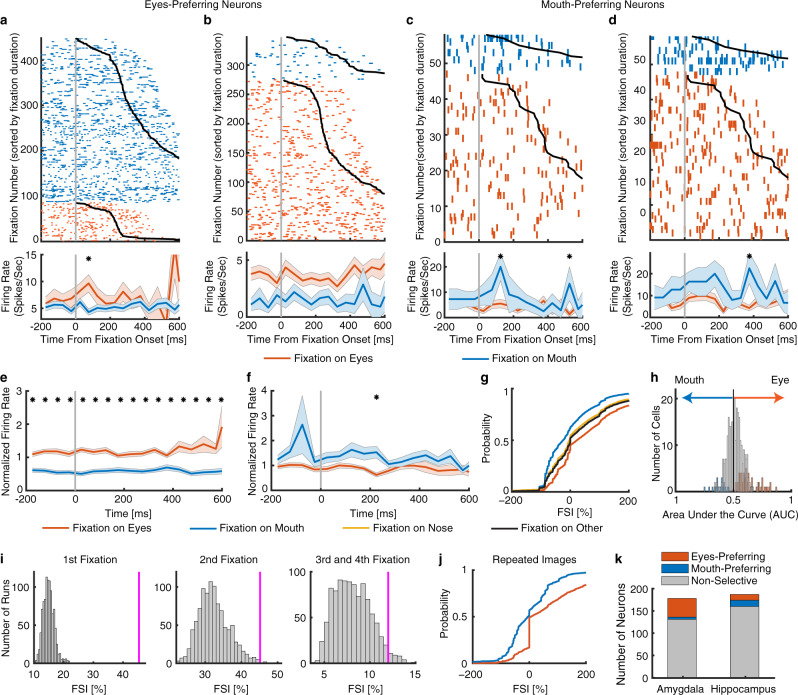
Neurons that differentiate fixations on the eyes vs. the mouth. **a**–**d** Fixation-aligned individual examples. **a, b** Neurons that had a greater firing rate when fixating on the eyes compared to the mouth (selection by two-tailed *t* test in a time window of −200 ms before fixation onset to 200 ms after fixation offset: both *P*s < 0.001). **c**, **d** Neurons that had a greater firing rate when fixating on the mouth compared to the eyes (both *P*s < 0.01). Fixations are sorted by fixation duration (black line shows start of the next saccade). Fixation onset is *t* = 0. Asterisk indicates a significant difference between fixations on the eyes and mouth in that bin (*P* < 0.05, two-tailed *t* test, after Bonferroni correction for multiple comparisons; bin size = 50 ms). Note that the selection of feature-selective neurons was based on the entire time window. **e**–**j** Population summary of all feature-selective neurons. **e** Average normalized firing rate of eyes-preferring neurons (*n* = 55). **f** Average normalized firing rate of mouth-preferring neurons (*n =* 19). Shaded area denotes ±SEM across neurons. Asterisk indicates a significant difference between the conditions in that bin (*P* < 0.05, two-tailed *t* test, after Bonferroni correction). **g** Single-fixation analysis using the fixation-selectivity index (FSI; Methods). Shown is the cumulative distribution of the single-fixation response of fixation-aligned eyes- and mouth-preferring neurons for fixations on the eyes and mouth (*n* = 74 neurons). **h** Population summary usi*n*g ROC analysis. Shown are histograms of AUC values of eyes-preferring neurons (red), mouth-preferring neurons (blue), and neurons that are neither eyes-preferring nor mouth-preferring (gray). **i** The FSI for fixation serial order. The magenta line indicates the observed mean FSI. The null distribution of the mean FSI (shown in gray histogram) was calculated by permutation tests of shuffling the labels of fixations on the eyes and mouth (1000 runs). **j** Cumulative distribution of the FSI for one-back repeated images. **k** The number of neurons in the amygdala and hippocampus from which recordings were made. Stacked bar shows eyes-preferring neurons (red), mouth-preferring neurons (blue), and nonfeature-selective neurons (gray).

To investigate the neural encoding of facial features, we first analyzed the response of each neuron between fixations on the eyes and mouth. We aligned neuronal responses at fixation onset and used the mean firing rate in a time window starting 200 ms before fixation onset and ending 200 ms after fixation offset (next saccade onset) to calculate statistics. The duration of this window was on average 814.7  ±  105.8 ms (mean  ±  SD across sessions). We identified 74/365 neurons (20.27%; binomial *P* < 10^−20^; see Supplementary Table 1 for a breakdown of each individual session) that had a response differing significantly between fixations on the eyes vs. the mouth (two-tailed *t* test, *P* < 0.05). We identified two types of such feature-selective neurons: one type had a greater response to the eyes relative to the mouth (“eyes-preferring”; 55/74 neurons [74%]; see Fig. 2a, b for individual examples and Fig. 2e for group results) and the second type had a greater response to the mouth relative to the eyes (“mouth-preferring”; 19/74 neurons [26%]; see Fig. 2c, d for individual examples and Fig. 2f for group results; note that the selection of feature-selective neurons was based on the entire time window; see also temporal dynamics in Fig. 2a–d [Bonferroni-corrected for multiple comparisons across time points]). The significant difference before fixation onset (Fig. 2a–f) indicated that feature selectivity had already started during preceding saccades, consistent with our prior findings^21,34^.

To investigate the relationship between the response of these feature-selective neurons and their behavior, we quantified the response of feature-selective neurons during individual fixations using a fixation-selectivity index (FSI; Methods). The FSI quantifies the response during each fixation relative to the mean response to fixations on the mouth and baseline, and it enables an aggregate measure of activity that pools eyes-preferring and mouth-preferring neurons by making the *average* FSI of a *neuron* positive for both types of neurons (see Eq. 1 and Eq. 2; note that the FSI for a *fixation* can be either positive or negative). By definition, for both eyes-preferring and mouth-preferring neurons, the mean FSI for *fixations on the mouth* will be equal to zero because the FSI is relative to the response to the fixation on the mouth, whereas the average value of FSI for *fixations on the eyes* will be greater than zero. Indeed, we observed that the distribution of the FSI for fixations on the mouth (shown by the cumulative distribution function [CDF] pooling fixations from both eyes-preferring and mouth-preferring neurons) was centered around 0 whereas the distribution of the FSI for fixations on the eyes was above 0 (i.e., the CDF shifted to the right; Fig. 2g). As expected, the FSI for feature-selective neurons was significantly larger during fixations on the eyes compared to fixations on the mouth (two-tailed two-sample Kolmogorov-Smirnov [KS] test, KS = 0.26, *P* < 10^−20^; Fig. 2g). This confirms that the single-fixation response of feature-selective neurons is strong enough to allow single-fixation analysis (see Fig. 2h for ROC analysis). Permutation tests were performed by shuffling the labels for the eyes and mouth, which further confirmed our results: feature-selective neurons (48.15 ± 5.41%, mean  ±  SD across neurons) had a significantly higher FSI compared to chance (13.90% ± 1.54%, permutation *P* < 0.001), whereas the FSI of all nonfeature-selective neurons (20.2 ± 1.49%) was not significantly above chance (19.50 ± 1.35%, permutation *P* = 0.28). Furthermore, we found that feature-selective neurons not only differentiated between the eyes vs. the mouth but also differentiated the eyes and mouth from other facial parts (i.e., the “nose” and “other” [all other areas of the entire image, including hair, neck, background, etc.] ROIs; KS-test: all *P*s < 10^−11^; Fig. 2g), suggesting that feature-selective neurons were specifically tuned for the eyes and mouth. Note that feature-selective neurons differentiated the eyes and mouth from other facial parts with a reduced strength (i.e., the CDFs of the nose and other were in between the CDFs of the eyes and the mouth).

Together, our results have shown that a subset of the amygdala and hippocampal neurons encode facial features by discriminating fixations onto the eyes vs. the mouth.

### Control analyses for feature selectivity

Because participants started viewing the faces from the image center given a preceding central fixation cross, the serial order of fixation onto each ROI might confound feature-selectivity and stimulus-evoked neuronal response (i.e., stimulus-evoked response might result in a greater response for later fixations). In a control analysis of fixation serial order, we found that feature-selective neurons still had a significantly above-chance FSI using the first fixation (45.37 ± 47.84%, permutation *P* < 0.001), second fixation (45.14 ± 67.73%, permutation *P* = 0.005), and combined third and fourth fixations (12.00 ± 29.96%, permutation *P* = 0.033; Fig. 2i) onto the eyes vs. the mouth alone, suggesting that our observed feature-selectivity could not be attributed to fixation serial order and stimulus-evoked neuronal response. In a second control analysis of image novelty, we found that feature-selective neurons still discriminated fixations onto the eyes vs. the mouth in the one-back repeated images (KS-test: KS = 0.34, *P* < 10^−20^; Fig. 2j), suggesting that face feature selectivity did not depend on image novelty. Lastly, the difference preceding fixation onset (Fig. 2e, f) was likely due to saccade planning and multiple fixations in the same ROI. However, we found qualitatively the same results when using different time windows to select feature-selective neurons (e.g., excluding time intervals before and/or after fixations).

**Fig. 3:**
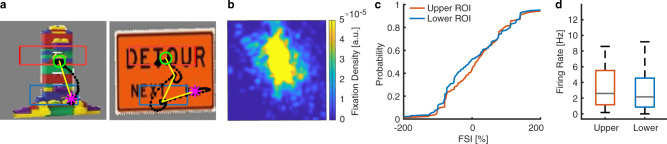
A control experiment using non-face stimuli. **a** Sample non-face stimuli. Each yellow dot represents a fixation. Green circle: first fixation. Magenta asterisk: last fixation. Yellow line: saccades. Black dot: raw gaze position. The upper (red box) and lower (blue box) regions of interest (ROIs) were derived from the CelebA stimuli using the average eyes and mouth ROIs, respectively. **b** The fixation density map. The map shows the probability of fixating a given location within the entire stimulus period. The scale bar (color bar) is in arbitrary units [a.u.]. **c** The cumulative distribution of the FSI. **d** Boxplot of the mean firing rate for fixations in the upper (red) vs. lower (blue) ROI (*n* = 19 neurons). On each box, the central mark is the median, the edges of the box are the 25th and 75th percentiles, and the whiskers extend to the most extreme data points the algorithm considers to be not outliers.

Could the response of feature-selective neurons be simply explained by upper vs. lower fixation locations? To address this important question, we recorded the response of a subset of the same 19 neurons using the same task but non-face stimuli (including furniture, toys, food, etc^21,35^; see Fig. 3a for examples and Fig. 3b for the average density map across all trials), and therefore, we were able to directly investigate whether feature selectivity was specific to faces. With the average eyes and mouth ROIs derived from the CelebA stimuli (Fig. 3a), we found that the FSI did not differ between fixations in the upper ROI (i.e., the average eyes ROI from the CelebA stimuli) vs. the lower ROI (i.e., the average mouth ROI from the CelebA stimuli; FSI: 17.91 ± 17.17%, permutation *P* = 0.84; KS = 0.11, *P* > 0.05; Fig. 3c), suggesting that feature-selective neurons did not simply encode upper vs. lower fixation locations but had specificity to faces. Furthermore, we found that the firing rate was similar between fixations on the upper vs. lower ROI (paired *t* test: *P* = 0.64, *t*(18) = 0.48; Fig. 3d), again confirming that the response of feature-selective neurons could not simply be explained by upper vs. lower fixation locations.

Lastly, do low-level visual features such as luminance explain facial feature selectivity? Although the luminance for the eyes (121.63  ±  26.43; mean  ±  SD across images) was lower than the luminance for the mouth (133.01  ±  29.94; two-tailed paired *t* test: *t*(499) = 13.80, *P* = 9.87×10^−37^) as expected, we found that the response of feature-selective neurons did not correlate with the luminance within the eyes ROI (Pearson’s *r* = 0.02  ±  0.11; mean  ±  SD across neurons; two-tailed paired *t* test against 0: *t*(73) = 1.52, *P* = 0.13), mouth ROI (*r* = 0.02  ±  0.19; *t*(69) = 0.93, *P* = 0.35), or regions outside the eyes and mouth ROIs (*r* = 0.006  ±  0.07; *t*(73) = 0.78, *P* = 0.44; note that the correlation had to be performed within the eyes or the mouth ROI because the difference between the eyes and mouth ROIs in both luminance and neural response would confound the correlation), suggesting that feature-selective neurons did not encode luminance per se.

Together, our control analyses have shown that face feature selectivity could not be attributed to fixation serial order, image novelty, or fixation locations.

### Comparison of cell types between eyes-preferring and mouth-preferring neurons

**Fig. 4:**
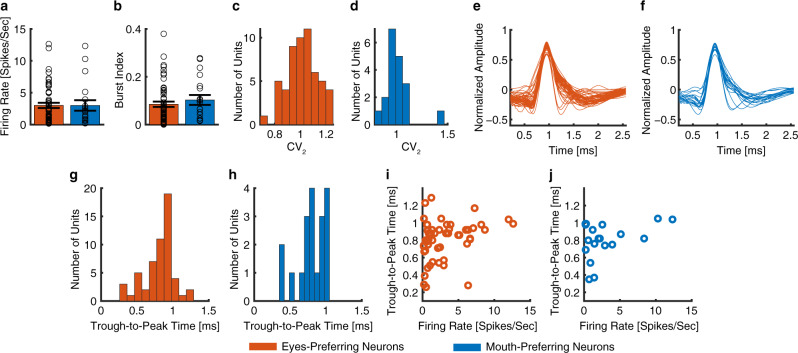
Comparison of cell type between eyes-preferring neurons and mouth-preferring neurons. **a** Mean firing rate. Error bar denotes ±SEM across neurons and circles show individual values. Red: eyes-preferring neurons. Blue: mouth-preferring neurons. **b** Burst index (BI). **c**, **e**, **g**, **i** Eyes-preferring neurons (*n* = 55). **d**, **f**, **h**, **j** Mouth-preferring neurons (*n* = 19). **c**, **d** Distribution of the modified coefficient-of-variation (CV_2_). **e**, **f** Mean action potential waveforms. **g**, **h** Distribution of trough-to-peak times. **i**, **j** Correlation between mean firing rate and trough-to-peak time. Neither eyes-preferring neurons nor mouth-preferring neurons showed a significant correlation.

It is worth noting that for both eyes-preferring neurons and mouth-preferring neurons, the difference was primarily driven by fixations on the mouth: eyes-preferring neurons had a decreasing firing rate for fixations on the mouth while mouth-preferring neurons had an increasing firing rate for fixations on the mouth, whereas the firing rate for fixations on the eyes remained relatively constant throughout the fixation period for both eyes-preferring (Fig. 2e) and mouth-preferring neurons (Fig. 2f). Given this different pattern of modulation, we next tested whether the electrophysiological properties of eyes-preferring and mouth-preferring neurons differed, which might indicate different cell types. However, we found no statistically significant differences in mean firing rate (Fig. 4a; eyes-preferring: 3.00  ±  3.05 Hz (mean  ±  SD across neurons), mouth-preferring: 3.00  ±  3.58 Hz; two-tailed two-sample *t* test: *t*(72) = 0.008, *P* = 0.99) nor in the variability of spike times (see Methods), as quantified by the burst index (Fig. 4b; eyes-preferring: 0.09  ±  0.08, mouth-preferring: 0.10  ±  0.09; *t*(72) = 0.79, *P* = 0.43) and the modified coefficient-of-variation (CV_2_) (Fig. 4c, d; eyes-preferring: 1.02  ±  0.114, mouth-preferring: 01.01  ±  0.14; *t*(72) = 0.043, *P* = 0.97). Moreover, waveforms of eyes-preferring and mouth-preferring neurons did not differ significantly (Fig. 4e, f) and the trough-to-peak times were statistically indistinguishable (Fig. 4g, h; eyes-preferring: 0.81  ±  0.22 ms, mouth-preferring: 0.80  ±  0.20 ms; *t*(72) = 0.13, P = 0.90; KS-test: *P* = 0.97; proportion of neurons with trough-to-peak times > 0.5 ms: eyes-preferring: 50/55, mouth-preferring: 17/19; *χ*^2^-test: *P* = 0.85). Lastly, neither eyes-preferring (Fig. 4i; *r*(55) = 0.26, *P* = 0.050) nor mouth-preferring neurons (Fig. 4j; *r*(19) = 0.38, *P* = 0.11) showed a significant correlation between mean firing rate and waveform as quantified by trough-to-peak time. Together, the basic electrophysiological signatures suggest that eyes-preferring and mouth-preferring neurons were not of different neuronal types.

### Neurons that encoded saccades to the eyes and mouth

**Fig. 5:**
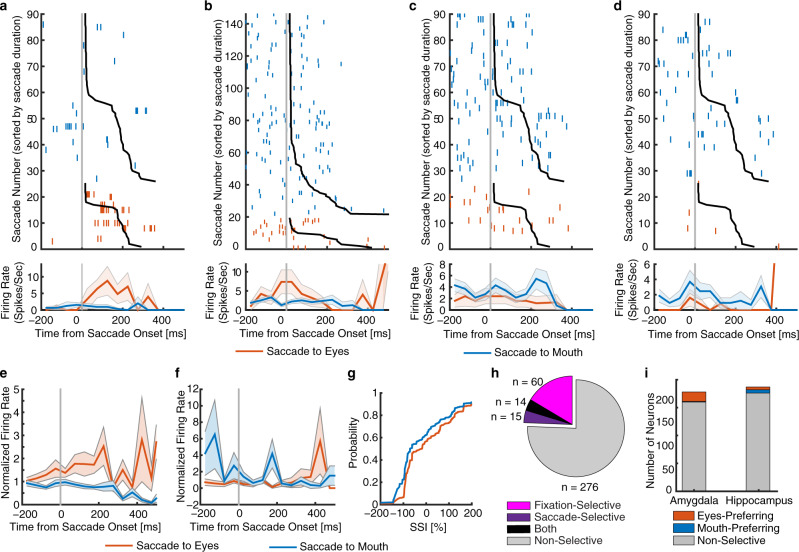
Neurons that encode saccades to eyes vs. mouth. **a**–**d** Saccade-aligned examples. **a**, **b** Neurons that had a greater firing rate when saccading to eyes compared to mouth (selection by two-tailed *t* test in a time window of −200 ms to 200 ms relative to saccade onset: both *P*s < 0.01). **c**, **d** Neurons that had a greater firing rate when saccading to mouth compared to eyes (both *P*s < 0.05). Saccades are sorted by saccade duration (black line shows start of the next fixation). *t* = 0 is saccade onset. Asterisk indicates a significant difference between saccades to the eyes/mouth vs. other facial parts in that bin (*P* < 0.05, two-tailed *t* test, after Bonferroni correction; bin size = 50 ms). **e**–**i** Population summary. **e** Average normalized firing rate of neurons that were selective to saccades to eyes (*n* = 22). **f** Average normalized firing rate of neurons that were selective to saccades to mouth (*n* = 7). Shaded area denotes ±SEM across neurons. Asterisk indicates a significant difference between the conditions in that bin (*P* < 0.05, two-tailed *t* test, after Bonferroni correction). **g** Single-saccade analysis using the SSI (Methods). Shown is the cumulative distribution of the single-saccade response. **h** Overlap between neurons showing fixation selectivity (magenta) and neurons showing saccade selectivity (purple). Black: both. Gray: neither. **i** The number of neurons in the amygdala and hippocampus. Stacked bar shows neurons that were selective to saccades to eyes (red), neurons that were selective to saccades to mouth (blue), and nonselective neurons (gray).

We next investigated whether neurons encoded saccades to the eyes or mouth. We aligned neuronal responses at saccade onset and used the mean firing rate in a time window from 200 ms before to 200 ms after saccade onset to calculate statistics. We identified 29 neurons (7.95%; binomial *P* = 0.006) that had a response differing significantly between saccades to the eyes vs. saccades to the mouth (two-tailed *t* test, *P* < 0.05). Among these neurons, 22 (75.9%) had a greater response for saccades to the eyes (see Fig. 5a, b for examples and Fig. 5e for group results) and 7 (24.1%) had a greater response for saccades to the mouth (see Fig. 5c, d for examples and Fig. 5f for group results). Similar to fixations, we quantified the response during individual saccades using a saccade-selectivity index (SSI; see Methods). As expected, the SSI for selective neurons was significantly larger during saccades to the eyes compared to saccades to the mouth (two-tailed two-sample KS-test, KS = 0.16, *P* = 8.88 × 10^−40^; Fig. 5g).

Furthermore, we found that 14/29 neurons (48.28%) that differentiated saccades to the eyes vs. the mouth were also feature-selective neurons (i.e., differentiated fixations onto the eyes vs. the mouth), showing a significantly higher percentage than the overall population (20.27%; *χ*^2^-test: *P* = 4.92 × 10^−4^; Fig. 5h). This suggests that saccade selectivity was related to subsequent fixation selectivity (similar results were derived when we established fixation selectivity without including the preceding saccade time interval: *P* = 6.73 × 10^−4^). Lastly, we derived similar results when we restricted our analysis within saccades between the eyes and mouth (i.e., excluding saccades initiated elsewhere), and we also derived similar results when we compared saccades to the eyes or mouth separately to all other saccades.

Together, we found a population of the amygdala and hippocampal neurons that encode saccade targets to the eyes and mouth. These neurons may elicit further responses of fixations onto the eyes and mouth.

### Population decoding

**Fig. 6:**
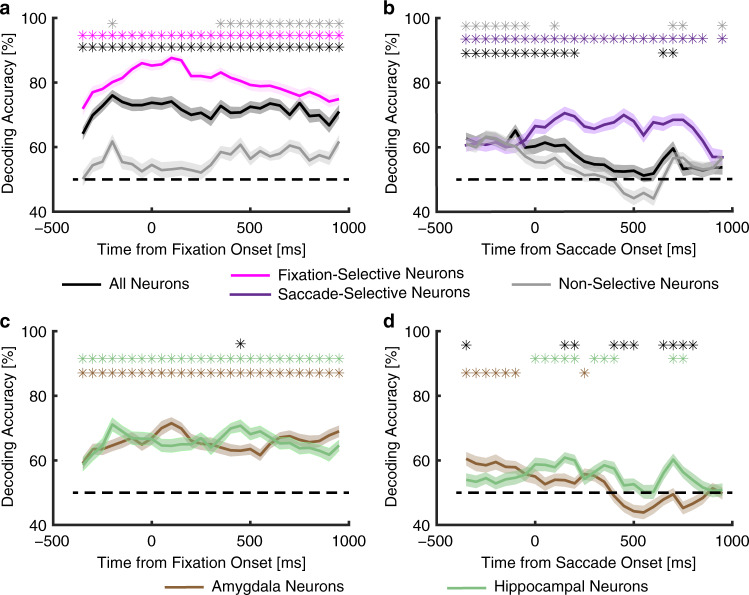
Neuronal population decoding. **a**, **c** Decoding of fixation on the eyes vs. the mouth. Bin size is 500 ms and step size is 50 ms. The first bin is from −600 ms to −100 ms (bin center: −350 ms) relative to fixation onset, and the last bin is from 700 ms to 1200 ms (bin center: 950 ms) after fixation onset. **b**, **d** Decoding of saccade to the eyes vs. mouth. Bin size is 500 ms and step size is 50 ms. The first bin is from −600 ms to −100 ms (bin center: −350 ms) relative to fixation onset, and the last bin is from 700 ms to 1200 ms (bin center: 950 ms) after fixation onset. **a**, **b** Decoding with all neurons (black), fixation-selective neurons (magenta), saccade-selective neurons (purple), and non-selective neurons (gray). Shaded area denotes ±SEM across bootstraps. The horizontal dashed black line indicates the chance level (50%). The top asterisks illustrate the time points with a significant above-chance decoding performance (paired *t* test against chance level, *P* < 0.05, corrected by FDR for *Q* < 0.05). **c**, **d** Decoding with all amygdala neurons (brown) and all hippocampal neurons (green). The top brown and green asterisks illustrate the time points with a significant above-chance decoding performance (paired *t* test against chance level, *P* < 0.05, corrected by FDR for *Q* < 0.05). The top black asterisks illustrate the time points with a significant difference between the amygdala and hippocampal neurons (two-tailed two-sample *t* test, *P* < 0.05, corrected by FDR for *Q* < 0.05).

How representative of the entire population of recorded neurons are the subsets of neurons described so far? In particular, although non-selective neurons could not distinguish between fixations on the eyes vs. the mouth or between saccades to the eyes vs. the mouth individually, could they still do so as a population? To answer these questions, we next employed population decoding. As expected, decoding from all recorded neurons together and decoding from feature-selective neurons revealed a strong ability to differentiate between fixations on the eyes vs. the mouth (Fig. 6a [magenta]; for all time bins). However, this ability was retained in nonfeature-selective neurons as well (Fig. 6a [gray]; primarily for later time bins), suggesting that nonfeature-selective neurons still carried information about differentiating facial features. Similarly, the whole population of neurons and those selective to saccade targets showed a strong ability to differentiate between saccades to the eyes vs. the mouth, but the non-selective neurons also partially retained this ability (Fig. 6b; primarily for early time bins).

### The amygdala and hippocampus played different roles in encoding facial features

Do the amygdala and hippocampus contribute equally to encoding facial features? Notably, we found that the amygdala (47/178 neurons; 26.4%) had a greater percentage of feature-selective neurons compared with the hippocampus (27/187 neurons; 14.4%; *χ*^2^ test: *P* = 0.0045; Fig. 2k; Supplementary Table 1). Furthermore, we found that among feature-selective neurons, the amygdala had a much greater percentage of eyes-preferring neurons (42/47 neurons; 89.4%) than the hippocampus (13/27 neurons; 48.2%; *χ*^2^ test: *P* = 9.35 × 10^−5^; Fig. 2k; Supplementary Table 1; and thus the amygdala had a lower percentage of mouth-preferring neurons). Therefore, the amygdala might play a different role in encoding facial features compared with the hippocampus, based on the greater percentage of feature-selective (in particular eyes-preferring) neurons in the amygdala. However, feature-selective neurons from the amygdala vs. hippocampus were not of different cell types (Supplementary Fig. 3).

Although the amygdala (18/178 neurons; 10.11%) did not have a significantly greater percentage of neurons that differentiated saccades to the eyes vs. the mouth than the hippocampus (11/187 neurons; 5.88%; *χ*^2^ test: *P* = 0.14; Fig. 5i), among these selective neurons, the amygdala (17/18 neurons; 94.4%) had a greater percentage of neurons that had a higher response to saccades to the eyes than those in the hippocampus (5/11 neurons; 45.5%; *χ*^2^ test: *P* = 0.0028; Fig. 5i).

Furthermore, both amygdala neurons and hippocampal neurons showed an above-chance decoding performance for fixation selectivity (Fig. 6c; for all time bins) and the decoding performance was similar between the amygdala and hippocampus (Fig. 6c). This confirms that both amygdala and hippocampal neurons contained information about fixations onto facial features. Interestingly, amygdala and hippocampal neurons had different decoding temporal dynamics when they encoded saccades (Fig. 6d): amygdala neurons tended to encode saccades earlier before saccade onset whereas the hippocampus tended to encode saccades later after saccade onset. This difference may indicate a hierarchal processing of saccades in the human medial temporal lobe.

### Feature-selective neurons encoded the saliency of facial features for face identification

**Fig. 7:**
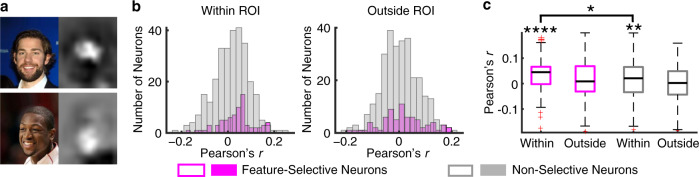
Feature-selective neurons encoded the saliency of facial features for face identification. **a** Two-sample face stimuli (left) and their saliency maps (right). **b** Distribution of correlation coefficients (Pearson’s *r*) across neurons. Correlation was calculated between the firing rate and saliency value across fixations for each neuron. The ROI combined both the eyes and the mouth. **c** Neuronal population average within and outside of the ROI. On each box, the central mark is the median, the edges of the box are the 25th and 75th percentiles, the whiskers extend to the most extreme data points the algorithm considers to be not outliers, and the outliers are plotted individually. Asterisks indicate a significant difference above 0 (two-tailed paired *t* test) or between feature-selective vs. non-selective neurons (two-tailed two-sample *t* test). *: *P* < 0.05, **: *P* < 0.01, and ****: *P* < 0.0001.

We next analyzed the functional consequence of feature-selective neurons. Using the VGG-16 deep neural network (DNN) trained for face recognition^36^, we constructed saliency maps of faces that represented critical features leading to face identification (i.e., the discriminative features of each face identity; e.g., the eyes [Fig. 7a upper] and the mouth [Fig. 7a lower]). We then correlated the saliency value under each fixation with the corresponding neuronal firing rate for each neuron (Fig. 7b). We found that feature-selective neurons showed a significantly above-chance (0) correlation for fixations within the preferred ROIs (i.e., within eyes and mouth; Pearson’s *r* = 0.04  ±  0.07; mean  ±  SD across neurons; two-tailed paired *t* test against 0: *t*(73) = 4.28, P = 5.52 × 10^−5^) but not for fixations outside the preferred ROIs (*r* = 0.015  ±  0.08; *t*(73) = 1.51, *P* = 0.13; Fig. 7c). Note that saliency was analyzed separately within or outside the eyes/mouth ROIs because these ROIs tended to have a higher saliency value (e.g., Fig. 7a; in other words, eyes/mouth contained more information about face recognition) and feature-selective neurons could discriminate eyes/mouth from other facial parts (Fig. 2g). Furthermore, feature-selective neurons showed a greater correlation with saliency values than nonfeature-selective neurons for fixations within the eyes/mouth ROIs (Fig. 7c; *t*(363) = 2.25, *P* = 0.025) but not for fixations outside the eyes/mouth ROIs (*t*(363) = 1.18, *P* = 0.24). Together, our results suggest that feature selectivity of eyes vs. mouth is related to face recognition, a possible functional consequence of feature-selective neurons.

### Feature-selective neurons were related to perceived social trait judgments

We lastly analyzed whether feature-selective neurons were related to implicit judgments of social traits. Because patients passively viewed the faces without providing explicit judgments of the faces during neural recordings, social traits might be implicitly processed in the brain. We subsequently acquired explicit ratings on a subset of faces (2–5 faces for each identity) through online questionnaires after patients were discharged. We acquired ratings for the following 8 social traits^37^: *warm, critical, competent, practical, feminine, strong, youthful*, and *charismatic*, and we used the average rating across patients for each face to calculate correlations. Note that these social traits were derived from a recent study that most comprehensively characterized trait judgments of faces using large-scale, preregistered online ratings^37^.

**Fig. 8:**
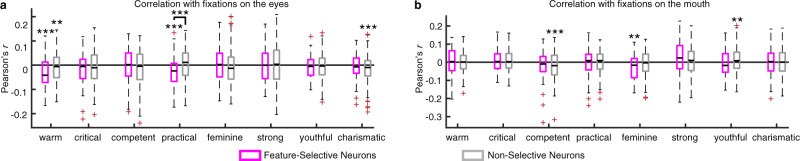
Feature-selective neurons were related to social traits. **a** Correlation between the firing rate for fixations on the eyes and perceived social traits. **b** Correlation between the firing rate for fixations on the mouth and perceived social traits. On each box, the central mark is the median, the edges of the box are the 25th and 75th percentiles, the whiskers extend to the most extreme data points the algorithm considers to be not outliers, and the outliers are plotted individually. Asterisks indicate a significant difference above 0 (two-tailed paired *t* test) or between feature-selective vs. non-selective neurons (two-tailed two-sample *t* test) after Bonferroni correction for multiple comparisons. **: *P* < 0.01 and ***: *P* < 0.001.

We found that the firing rate for fixations on the eyes significantly correlated with the social trait of *warm*, for both feature-selective neurons (Pearson’s *r* = −0.03  ±  0.06; mean  ±  SD across neurons; two-tailed paired *t* test against 0: *t*(73) = 4.05, *P* = 0.001; Bonferroni correction; Fig. 8a) and nonfeature-selective neurons (*r* = −0.011  ±  0.06; *t*(288) = 3.23, *P* = 0.0014). Similarly, the firing rate for fixations on the eyes significantly correlated with the social trait of *practical* for feature-selective neurons (*r* = −0.028  ±  0.066; *t*(73) = 3.60, *P* = 5.84 × 10^−4^) but not nonfeature-selective neurons (*r* = 0.003  ±  0.064; *t*(288) = 0.77, *P* = 0.44; Fig. 8a), and feature-selective neurons showed a significantly stronger correlation (*t*(361) = 3.64, *P* = 3.13×10^−4^). Furthermore, we found that the firing rate for fixations on the mouth significantly correlated with the social trait of *feminine* for feature-selective neurons (*r* = −0.03  ±  0.07; *t*(64) = 2.99, *P* = 0.004) but not nonfeature-selective neurons (*r* = 0.006  ±  0.06; *t*(273) = 1.45, *P* = 0.15; Fig. 8b). Notably, the above results were primarily driven by amygdala neurons (Supplementary Fig. 4); and the stronger correlations between neuronal responses and social trait judgments in the amygdala was consistent with human lesion studies^38^. Note that patients showed consistent ratings for most of the traits, and similar results were derived using each patient’s own ratings. Also note that here both positive and negative correlations were meaningful because the description of a social trait could be bidirectional (e.g., a positive correlation with *warm* was equivalent to a negative correlation with *cold*).

Together, we found that the neuronal response to facial features was related to the perception of social traits and feature-selective neurons were more related to social traits than nonfeature-selective neurons.

## Discussion

In this study, we recorded neural activity from the amygdala and hippocampal neurons in neurosurgical patients using implanted depth electrodes while they viewed static images of famous faces. We identified the neural mechanisms underlying human visual scanning patterns of faces by revealing a class of neurons sensitive to different facial features (the eyes and mouth). We found that the amygdala had a greater percentage of feature-selective neurons that were eyes-preferring compared with the hippocampus, although feature-selective neurons from the amygdala vs. hippocampus and eyes-preferring vs. mouth-preferring neurons were not of different neuronal types. We further revealed neurons that were sensitive to saccade targets and we confirmed these results using population decoding. Lastly, we revealed the functional roles of feature-selective neurons. Feature-selective neurons encoded the saliency of facial features so they may contribute to face identification and feature-selective neurons were correlated with perceived social traits so they may also contribute to face evaluation of social judgments. Together, we not only characterized a class of neurons that encoded facial features, but also suggest they may play important roles in face recognition and judgment.

Previous neuroimaging studies have shown that the amygdala is sensitive to facial features and saccade directions^28^, consistent with our present findings. These neuroimaging studies varied locations of the faces on the screen for each trial (either aligning the eyes or mouth to the screen center) in order to control for fixation starting location and fixation sequence. In the present study, our single-neuron recordings had a superior temporal resolution and our simultaneous eye-tracking allowed fixation-based analysis (rather than trial-based analysis found in neuroimaging studies). We have further shown that our observed feature selectivity could not be explained by fixation serial order (Fig. 2i). Furthermore, compared with previous research, our findings were derived using a large variety of natural face images; and we have replicated our findings using separate tasks and a new set of cartoon faces (manuscript in preparation). In addition, we have extended previous research showing that amygdala neurons are selective to whole vs. piecemeal faces^29^ by further showing that amygdala and hippocampal neurons are selective to fixations and saccades to salient facial features such as the eyes and mouth. Our results were also consistent with the role of the hippocampus in encoding saccade directions^39^. Notably, in the present study we observed responses occurring before fixation onset (Fig. 2b, e, f), which is consistent with both previous human single-neuron studies^21,34^ and human intracranial event-related potential (ERP) studies^40^.

We found feature-selective neurons in both amygdala and hippocampus but the amygdala had a higher percentage of feature-selective neurons and in particular eyes-preferring neurons than the hippocampus, which highlighted different roles between the amygdala and hippocampus in encoding facial features. Such difference in function between the amygdala and hippocampus is consistent with our previous finding showing that only the amygdala but not the hippocampus encodes perceived facial emotions^19^. However, the amygdala and hippocampus play a similar role in encoding face identities^41^, visual selectivity^14,21^, memory^42^, and visual attention^21^. Given that an amygdala lesion abolishes saccading to the eyes^10^ but not aspects of visual attention^35,43,44^ whereas a hippocampal lesion impairs visual search and exploration^45–47^, a future study will be needed to investigate whether the hippocampus plays a necessary role in facial feature selectivity.

Although some faces in the CelebA stimuli have facial expressions (because the celebrities posed when taking the photos) and eye movement can be influenced by facial expressions^48^, we observed similar results using emotionally neutral FaceGen model faces. Therefore, our results could not simply be explained by the neuronal response to facial emotions^19,20^. Furthermore, patients provided social judgment ratings after they were discharged and we used patients’ average ratings to correlate with the neuronal responses. On the one hand, although patients provided ratings in a different environment compared to the recordings (home vs. hospital) and the social judgment ratings might change over time, it has been shown that judging the social traits used in the present study is in general reliable and has a good within-subject consistency^37^ (also note that a social trait is a temporally stable characteristic). Notably, it was our intention and design to unpair recordings and ratings because we were probing the neural mechanisms underlying *implicit* judgments of social traits. On the other hand, social judgment may be variable across individuals. However, we derived similar results when we used each patient’s own ratings for analysis (patients showed consistent ratings for most of the traits), and we also derived similar results when we used consensus ratings from a large number of online participants for analysis (patients’ ratings were also generally consistent with the consensus ratings).

Monkey studies have shown that amygdala neurons demonstrate levels of nested selectivity (e.g., face-selective neurons show additional selectivity for individuals, and these identity-selective neurons further differentiate between the facial expressions of that particular individual)^25,31^. However, in contrast to such nested selectivity for monkey facial feature-selective neurons^31^, the facial feature-selective neurons observed in the present study did not depend on face responsiveness (only 14.55% of face-responsive neurons were feature-selective neurons, a percentage that was even lower than the entire population [20.27%]) or identity selectivity (only 13.21% of identity-selective neurons were feature-selective neurons). Furthermore, we found that eyes-preferring neurons (but not mouth-preferring neurons) only showed a marginal effect for eye contact (i.e., contrasting between fixations on eyes with direct vs. averted gazes). These differences may be due to the use of dynamic^31^ vs. static stimuli; and a future comparative study will be needed to systematically understand the difference between human and monkey facial feature neurons (e.g.,^49^).

Because the eyes and mouth are the most salient and informative facial features, our results support the idea that the amygdala encodes socially relevant and salient stimuli^27^. Furthermore, our results are in line with findings from lesion studies showing that a focal amygdala lesion abolishes orienting to the eyes of fearful faces^10^, as well as findings from autism research showing that decreased orientation to the eyes in autism is attributed to reduced neural response in the amygdala^30,50^. It is worth noting that the latter studies either varied initial face location^50^ or used unnatural piecemeal “bubbles” stimuli^30^ in order to study the neural response to facial features, while our present study employed concurrent neural recordings and eye tracking that allowed us to directly probe the neural response as a function of eye movement to facial features in the natural viewing of faces. Lastly, although a face feature space has been observed in nonhuman primates^51^, our present study not only directly showed facial feature selectively in humans at the fixation/saccade level but also revealed possible functional roles of facial feature selectivity for face identification and evaluation. This research significantly deepens our understanding of the neural underpinnings of visual processing of human faces, which is critical for future investigations into disorders where face processing goes awry.

## Methods

### Participants

There were 16 sessions from five patients in total. All sessions had simultaneous eye tracking. We excluded 4 sessions that had fewer than 10 fixations onto each facial region of interest (ROI) due to a substantial amount of missing eye-tracking data, resulting in a total of 12 sessions for further analysis (Supplementary Table 1). All participants provided written informed consent using procedures approved by the Institutional Review Board of the West Virginia University (WVU).

### Stimuli

We used faces of celebrities from the CelebA dataset^52^. We selected 50 identities with 10 images for each identity, totaling 500 face images. The identities were selected to include both genders and multiple races. We used the same stimuli for all patients. Patients were asked to indicate whether they were familiar with each identity in a follow-up survey.

Patients were also asked to provide judgments of social traits on a one to seven scale through an online questionnaire after they were discharged. The social traits include *warm, critical, competent, practical, feminine, strong, youthful*, and *charismatic*. Three patients completed the questionnaire and depending on the availability of the patients, patients provided ratings for two to five faces per identity per social trait (the rated faces were all from the original stimuli). We observed high inter-subject consistency so we used the average rating for each face to correlate perceptions of social judgment with neuronal firing rate. We also included neuronal data from the two patients who did not provide ratings.

### Experimental procedure

We used a simple 1-back task. In each trial, a single face was presented at the center of the screen for a fixed duration of 1 s, with uniformly jittered inter-stimulus-interval (ISI) of 0.5–0.75 s (Fig. 1a). Each image subtended a visual angle of approximately 10°. Patients pressed a button if the present face image was *identical* to the immediately previous image. 9% of trials were one-back repetitions. Each face was shown once unless repeated in one-back trials and we excluded responses from one-back trials to have an equal number of responses for each face. This task kept patients attending to the faces, but avoided potential biases from focusing on a particular facial feature (e.g., compared to asking patients to judge a particular facial feature). The order of faces was randomized for each patient. This task procedure has been shown to be effective to study face representation in humans^53^.

Stimuli were presented using MATLAB with the Psychtoolbox 3^54^ (http://psychtoolbox.org) (screen resolution: 1600 × 1280).

### Eye tracking

Patients were recorded with a remote non-invasive infrared Eyelink 1000 system (SR Research, Canada). One of the eyes was tracked at 500 Hz. The eye tracker was calibrated with the built-in 9-point grid method at the beginning of each block. Fixation extraction was carried out using software supplied with the Eyelink eye-tracking system. Saccade detection required a deflection of greater than 0.1°, with a minimum velocity of 30°/s and a minimum acceleration of 8000°/s^2^, maintained for at least 4 ms. Fixations were defined as the complement of a saccade, i.e., periods without saccades. Analysis of the eye movement record was carried out off-line after the completion of the experiments.

### Electrophysiology

We recorded from implanted depth electrodes in the amygdala and hippocampus from patients with pharmacologically intractable epilepsy. Target locations in the amygdala and hippocampus were verified using post-implantation CT. At each site, we recorded from eight 40 μm microwires inserted into a clinical electrode as described previously^42,55^. Efforts were always made to avoid passing the electrode through a sulcus, and its attendant sulcal blood vessels, and thus the location varied but was always well within the body of the targeted area. Microwires projected medially out at the end of the depth electrode and examination of the microwires after removal suggests a spread of about 20–30°. The amygdala electrodes were likely sampling neurons in the mid-medial part of the amygdala and the most likely microwire location is the basomedial nucleus or possibly the deepest part of the basolateral nucleus. Bipolar wide-band recordings (0.1–9000 Hz), using one of the eight microwires as the reference, were sampled at 32 kHz and stored continuously for off-line analysis with a Neuralynx system. The raw signal was filtered with zero-phase lag 300–3000 Hz bandpass filter and spikes were sorted using a semi-automatic template matching algorithm as described previously^56^. Units were carefully isolated and recording and spike sorting quality were assessed quantitatively (Supplementary Fig. 2). Only units with an average firing rate of at least 0.15 Hz (entire task) were considered^41^.

### Response index for single fixation or saccade

For each neuron, we quantified whether its response differed between a fixation on the eyes and fixation on the mouth using a single-fixation selectivity index, FSI (Eq. 1 and Eq. 2). The FSI facilitates group analysis and comparisons between different types of cells (i.e., eyes- and mouth-preferring cells in this study), as motivated by previous studies^19,21^. The FSI quantifies the response during fixation *i* relative to the mean response to fixations on the mouth and baseline (the interval right before face onset). The mean response and baseline were calculated individually for each neuron.

1 $${{{{{\rm{Eyes}}}}}}-{{{{{\rm{preferring}}}}}}\!\!:{FS}{I}_{i}=\frac{F{R}_{i}-{mean}(F{R}_{{Mouth}})}{{mean}(F{R}_{{Baselin}e})}\cdot 100 \%$$

2 $${{{{{\rm{Mouth}}}}}}-{{{{{\rm{preferring}}}}}}\!\!:{FS}{I}_{i}=-\frac{F{R}_{i}-{mean}(F{R}_{{Mouth}})}{{mean}(F{R}_{{Baseline}})}\cdot 100 \%$$

For each fixation *i*, *FSI*_*i*_ is the baseline normalized mean firing rate (FR) during an interval from 200 ms before fixation onset to 200 ms after fixation offset (the same time interval as cell selection). Different time intervals were tested as well, to ensure that results were qualitatively the same and not biased by particular spike bins.

If a neuron distinguishes fixations on the eyes from fixations on the mouth, the mean value of FSI across all fixations will be significantly different from 0. Since eyes-preferring neurons have more spikes in fixations on the eyes, the mean FSI is positive for eyes-preferring neurons (Eq. 1). Since mouth-preferring neurons have more spikes in fixations on the mouth, to get an aggregate measure of activity that pools across neurons, *FSI*_*i*_ was multiplied by −1 if the *neuron* is classified as a mouth-preferring neuron (Eq. 2) so that the mean FSI is also positive for mouth-preferring neurons. Therefore, Eq. 1 and Eq. 2 make the mean FSI positive for both types of feature-selective neurons. Notice that the factor −1 in Eq. 2 depends only on the *neuron* type but not *fixation* type (note that each neuron type has both fixations on the eyes and fixations on the mouth).

After calculating *FSI*_*i*_ for every fixation, we subsequently averaged all *FSI*_*i*_ of fixations that belong to the same category (e.g., eyes, mouth, nose, and other). By definition, the average value of FSI for *fixations on the mouth* will be equal to zero because the definition of *FSI*_*i*_ is relative to the response to fixation on the mouth (see Eq. 1 and Eq. 2; see also Fig. 2g); and the average value of FSI for *fixations on the eyes* will be greater than zero. Notably, this is the case for both eyes-preferring neurons and mouth-preferring neurons (also note that each neuron type has both fixations on the eyes and fixations on the mouth). The mean baseline firing rate was calculated across all trials. The same *FR*_*Mouth*_ was subtracted for all types of fixations.

The cumulative distribution function (CDF) was constructed by calculating for each possible value *x* of the FSI how many examples are smaller than *x*. That is, F(*x*) = P(*X* ≤ *x*), where *X* is a vector of all FSI values. The CDFs of fixations of different categories were compared using two-tailed two-sample Kolmogorov-Smirnov (KS) tests.

Similarly, we defined single-saccade selectivity index (SSI) during an interval from 200 ms before to 200 ms after saccade onset (the same time interval as cell selection) as we quantified saccades to the eyes or mouth.

### Comparison of cell types

We quantified basic electrophysiological parameters following previous studies^30,57^. To compare the variability of spike times, we computed the inter-spike interval (ISI) distribution of each cell by considering all spikes fired during the experiment and quantified it using two metrics: the burst index (BI) and the modified coefficient-of-variation (CV_2_). The BI was defined as the proportion of ISIs less than 10 ms^58^. The CV_2_ (Eq. 3) is a function of the difference between two adjacent ISIs and is a standard measure to quantify spike-train variability that is robust to underlying rate changes^59^. In contrast, the coefficient-of-variation measure CV is only valid for stationary processes (i.e., fixed mean firing rate) and is thus not applicable for this analysis.

3 $$C{V}_{2}=\frac{1}{N}\mathop{\sum }\limits_{i=1}^{N}\frac{2|IS{I}_{i+1}-IS{I}_{i}|}{IS{I}_{i+1}+IS{I}_{i}}$$

We compared the waveform of different neurons based on the trough-to-peak time of the mean waveform^60^. The mean waveform is the average of all spikes assigned to the cluster. The polarity of the mean waveforms was inverted if necessary such that the trough always occurs before the peak. We also evaluated whether there was a correlation between the trough-to-peak time and the mean firing rate of a unit. For this, the mean firing rate was defined as the mean rate over the entire duration of all valid trials.

### Statistics and reproducibility

Fixations were aligned to fixation onset and saccades were aligned to saccade onset. Average firing rates Peri-Stimulus-Time Histogram (PSTH) were computed by counting spikes across all fixations or across all saccades in consecutive 50 ms bins. Pairwise comparisons were made using a two-tailed *t* test at *P* < 0.05 and Bonferroni-corrected for multiple comparisons in time bins in the group PSTH. Asterisks in the figures indicate a significant difference after the Bonferroni correction.

For single-neuron Receiver Operating Characteristic (ROC) analysis, we constructed ROC curves based on the spike counts in a time window of 200 ms before fixation onset to 200 ms after fixation offset. We varied the detection threshold between the minimal and maximal spike count observed, linearly spaced in 20 steps. The Area Under the Curve (AUC) of the ROC was calculated by integrating the area under the ROC curve (trapezoid rule). The AUC value is an unbiased estimate for the sensitivity of an ideal observer that counts spikes and makes a binary decision based on whether the number of spikes is above or below a threshold. We defined the category with a higher overall firing rate as ‘true positive’ and the category with a lower overall firing rate as ‘false positive’. Therefore, the AUC value was always above 0.5 by definition.

For neuronal population decoding of fixations, we pooled all recorded neurons into a large pseudo-population (see^22,34^). Firing rates were *z*-scored individually for each neuron to give equal weight to each unit regardless of the firing rate. We used a maximal correlation coefficient classifier (MCC) as implemented in the MATLAB neural decoding toolbox (NDT)^61^. The MCC estimates a mean template for each class *i* and assigns the class for test fixation. We used 8-fold cross-validation, i.e., all fixations were randomly partitioned into eight equal-sized subsamples, of which seven subsamples were used as the training data and the remaining single subsample was retained as the validation data for assessing the accuracy of the model, and this process was repeated eight times, with each of the 8 subsamples used exactly once as the validation data. We then repeated the cross-validation procedure 50 times for different random train/test splits. Statistical significance of the decoding performance for each group of neurons against chance was estimated by calculating the percentage of bootstrap runs (50 in total) that had an accuracy below chance (i.e., 50% when decoding the type of ROI). Statistical significance for comparing between groups of neurons was estimated by calculating the percentage of bootstrap runs (50 in total) that one group of neurons had a greater accuracy than the other. For both tests, we used false discovery rate (FDR)^62^ to correct for multiple comparisons across time points. Spikes were counted in bins of 500 ms and advanced by a step size of 50 ms. The first bin started −600 ms relative to fixation onset (bin center was thus 350 ms before fixation onset), and we tested 27 consecutive bins (the last bin was thus from 700 ms to 1200 ms after fixation onset). For each bin, a different classifier was trained/tested.

For neuronal population decoding of saccades, we also used 8-fold cross-validation and repeated the process 50 times with different subsets of saccades, resulting in a total of 400 tests to estimate the test performance. Spikes were counted in bins of 500 ms and advanced by a step size of 50 ms. The first bin started −600 ms relative to saccade onset (bin center was thus 350 ms before saccade onset), and we tested 27 consecutive bins (the last bin was thus from 700 ms to 1200 ms after saccade onset). For each bin, a different classifier was trained/tested.

### Reporting summary

Further information on research design is available in the Nature Research Reporting Summary linked to this article.

## Supplementary information

Supplementary Information

Description of Additional Supplementary Files

Supplementary Data 1

Supplementary Data 2

Supplementary Data 3

Supplementary Data 4

Supplementary Data 5

Supplementary Data 6

Supplementary Data 7

Supplementary Data 8

Supplementary Data 9

Supplementary Data 10

Supplementary Data 11

Reporting Summary

## Data Availability

The full data set for this study is publicly available on OSF (https://osf.io/6ekrz/). The source data to generate each figure is available from Supplementary Data 1–11.

## Code availability

The source code for this study is publicly available on OSF (https://osf.io/6ekrz/).
