# Supplementary material for: Encoding of facial features by single neurons in the human amygdala and hippocampus
Source: Commun Biol. 2021 Dec 14;4:1394. doi: 10.1038/s42003-021-02917-1 (PMC8671411; doi:10.1038/s42003-021-02917-1)
Supplement: Supplementary file 1 — Supplementary Information [file 42003_2021_2917_MOESM1_ESM.pdf]

Supplementary Materials

Supplementary Table 1. List of patients and sessions. Each row of neurons represents a separate recording session, which was recorded on a separate day. Total: all neurons recorded from an area. Left: neurons that were recorded from the left hemisphere and had a firing rate greater than 0.15 Hz. Right: neurons that were recorded from the right hemisphere and had a firing rate greater than 0.15 Hz. These neurons were included for further analysis. Eye: eyes-preferring feature-selective neurons. Mouth: mouth-preferring feature-selective neurons. Patient P8 was not included in the study because the patient only had surface electrodes.

ID	Age	Sex	Epilepsy diagnosis	Number of Amygdala Neurons					Number of Hippocampal Neurons				
				Total	Left	Right	Eye	Mouth	Total	Left	Right	Eye	Mouth
P6	33	F	Left posterior temporal/ parietal	9	9	0	0	0	31	31	0	1	2
				10	10	0	0	1	29	29	0	4	4
P7	28	F	Right mesial temporal	6	6	0	0	0	21	19	2	0	2
				6	6	0	1	1	20	19	1	1	0
				2	2	0	0	0	31	26	5	2	4
P9	42	M	Left frontal	28	28	0	4	3	7	7	0	2	1
				29	29	0	5	0	7	7	0	1	0
				25	25	0	10	0	6	6	0	0	0
				23	23	0	15	0	3	3	0	1	0
P10	47	F	Right temporal	25	0	25	2	0	7	7	0	1	0
P11	33	F	Right temporal	9	0	9	5	0	15	0	15	0	1
				6	0	6	0	0	10	0	10	0	0
Sum				178	138	40	42	5	187	154	33	13	14

Supplementary Figures

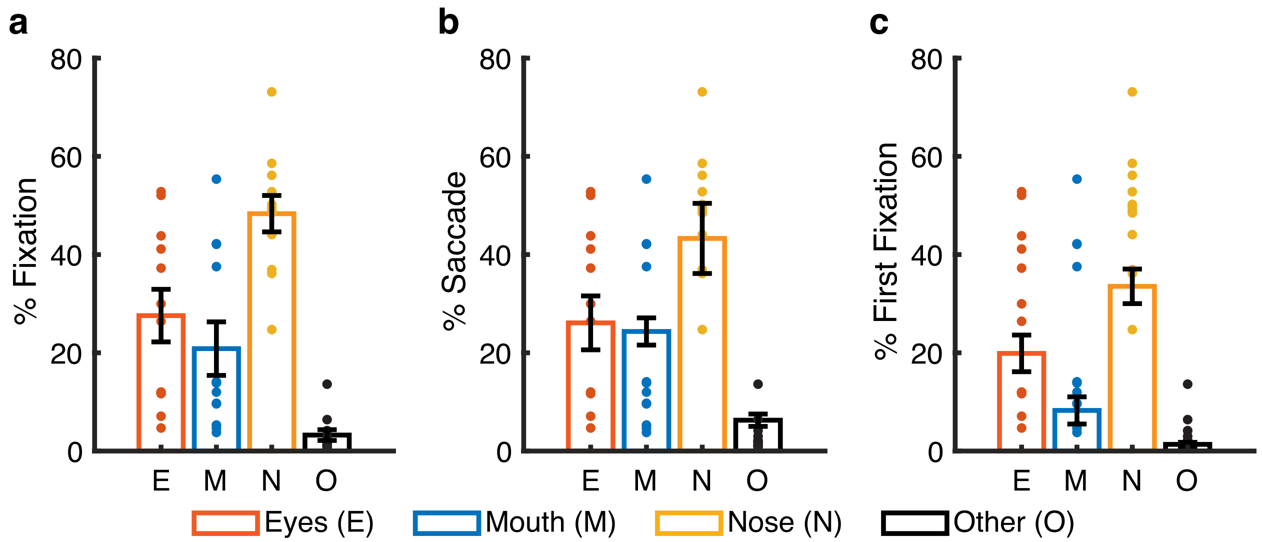

Supplementary Fig. 1. Percentage of fixation/saccade after normalization by the areas of regions of interest (ROIs). **(a)** Percentage of fixation for each ROI. **(b)** Percentage of saccade to each ROI. **(c)** Percentage of first fixation onto each ROI. Error bars denote ±SEM across sessions. Each dot represents an individual session. E: eyes. M: mouth. N: nose. O: other (all other parts of the image, including hair, neck, background, etc.).

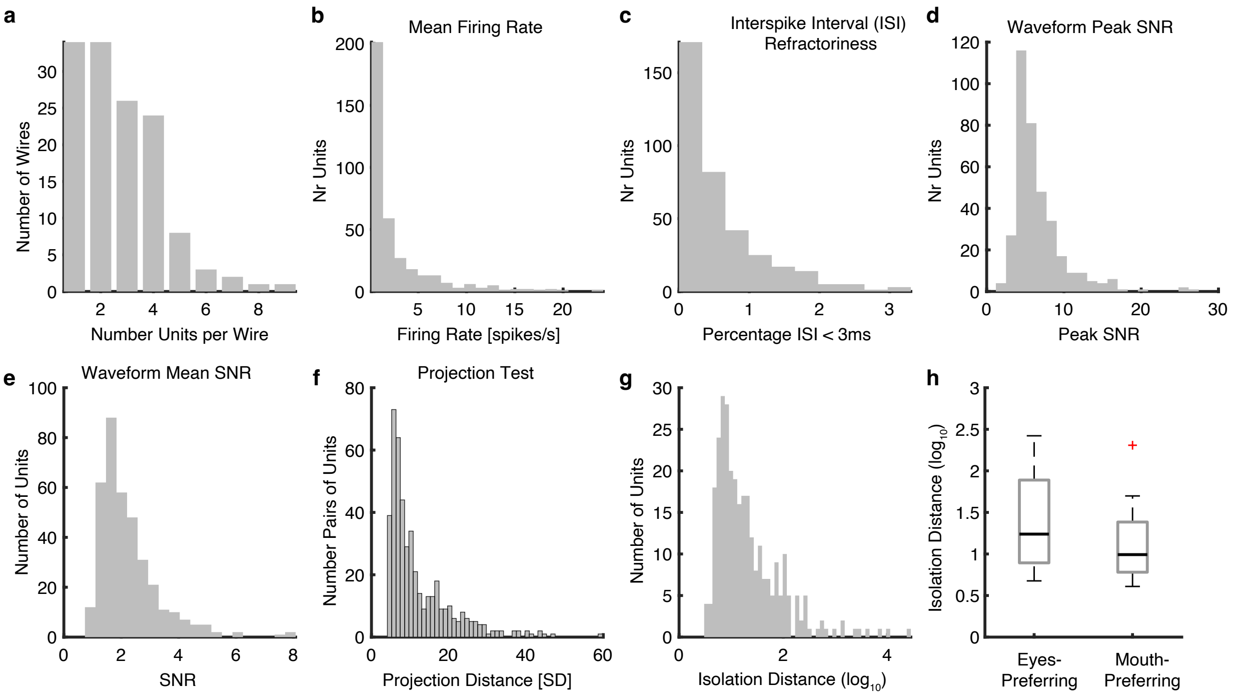

Supplementary Fig. 2. Spike sorting and recording quality assessment. **(a)** Histogram of the number of units identified on each active wire (only wires with at least one unit identified are counted). The average yield per wire with at least one unit was 2.74±1.60 (mean±SD). **(b)** Histogram of mean firing rates. **(c)** Histogram of proportion of inter-spike intervals (ISIs) which are shorter than 3 ms. The large majority of clusters had less than 0.5% of such short ISIs. **(d)** Histogram of the signal-to-noise ratio (SNR) of the mean waveform peak of each unit. **(e)** Histogram of the SNR of the entire waveform of all units. **(f)** Pairwise distance between all possible pairs of units on all wires where more than 1 cluster was isolated. Distances are expressed in units of standard deviation (SD) after normalizing the data such that the distribution of waveforms around their mean is equal to 1. **(g)** Isolation distance of all units for which this metric was defined (n = 365, median = 13.64). **(h)** Eyes-preferring and mouth-preferring neurons did not differ significantly in isolation distance ($t(60) = 1.55$, P = 0.13). On each box, the central mark is the median, the edges of the box are the 25th and 75th percentiles, the whiskers extend to the most extreme data points the algorithm considers to be not outliers, and the outliers are plotted individually.

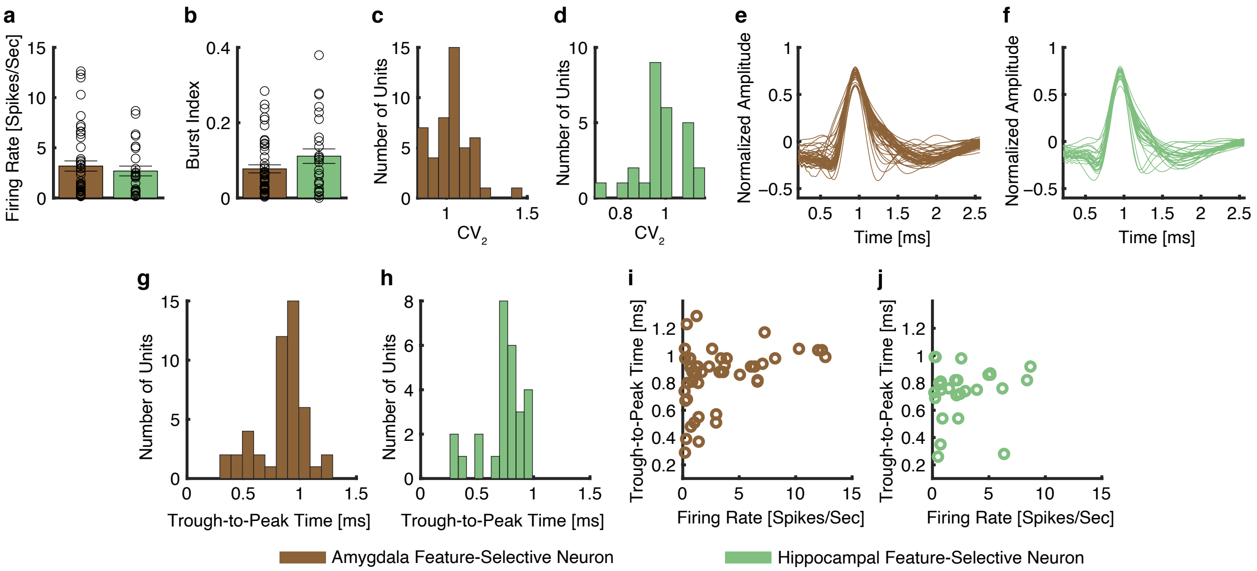

Supplementary Fig. 3. Comparison of cell types between amygdala vs. hippocampus feature-selective neurons. **(a)** Mean firing rate. Error bar denotes ±SEM across neurons and circles show individual values. Brown: amygdala feature-selective neurons. Green: hippocampus feature-selective neurons. **(b)** Burst index (BI). **(c, e, g, i)** Amygdala feature-selective neurons (n = 47). **(d, f, h, j)** Hippocampus feature-selective neurons (n = 27). **(c, d)** Distribution of the modified coefficient-of-variation (CV_2). **(e, f)** Mean action potential waveforms. **(g, h)** Distribution of trough-to-peak times. **(i, j)** Correlation between mean firing rate and trough-to-peak time.

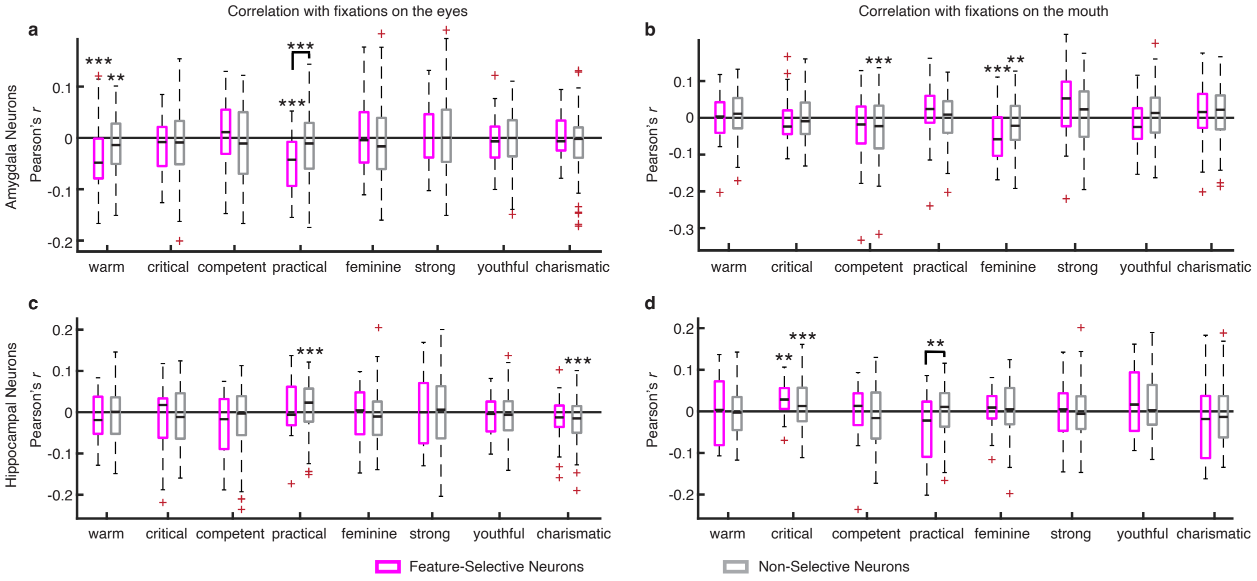

Supplementary Fig. 4. Separate analysis of amygdala and hippocampal neurons for encoding of social traits. **(a, b)** Amygdala neurons (n = 47 for feature-selective neurons and n = 131 for non-feature-selective neurons). **(c, d)** Hippocampal neurons (n = 27 for feature-selective neurons and n = 160 for non-feature-selective neurons). **(a, c)** Correlation between the firing rate for fixations on the eyes and perceived social traits. **(b, d)** Correlation between the firing rate for fixations on the mouth and perceived social traits. On each box, the central mark is the median, the edges of the box are the 25th and 75th percentiles, the whiskers extend to the most extreme data points the algorithm considers to be not outliers, and the outliers are plotted individually. Asterisks indicate a significant difference above 0 (two-tailed paired t-test) or between feature-selective vs. non-selective neurons (two-tailed two-sample t-test) after Bonferroni correction for multiple comparisons. **: $P < 0.01$ and ***: $P < 0.001$.
