# Supplementary material for: Encoding of facial features by single neurons in the human amygdala and hippocampus
Source: Commun Biol. 2021 Dec 14;4:1394. doi: 10.1038/s42003-021-02917-1 (PMC8671411; doi:10.1038/s42003-021-02917-1)
Supplement: Supplementary file 2 — Description of Additional Supplementary Files [file 42003_2021_2917_MOESM2_ESM.pdf]

Description of Additional Supplementary Files

File name: Supplementary Data 1
Description: Source data for generating Fig. 1

File name: Supplementary Data 2
Description: Source data for generating Fig. 2

File name: Supplementary Data 3
Description: Source data for generating Fig. 3

File name: Supplementary Data 4
Description: Source data for generating Fig. 4

File name: Supplementary Data 5
Description: Source data for generating Fig. 5

File name: Supplementary Data 6
Description: Source data for generating Fig. 6

File name: Supplementary Data 7
Description: Source data for generating Fig. 7

File name: Supplementary Data 8
Description: Source data for generating Fig. 8 and Supplementary Fig. 4.

File name: Supplementary Data 9
Description: Source data for generating Supplementary Fig. 1.

File name: Supplementary Data 10
Description: Source data for generating Supplementary Fig. 2.

File name: Supplementary Data 11
Description: Source data for generating Supplementary Fig. 3.
