# Supplementary material for: Encoding of facial features by single neurons in the human amygdala and hippocampus
Source: Commun Biol. 2021 Dec 14;4:1394. doi: 10.1038/s42003-021-02917-1 (PMC8671411; doi:10.1038/s42003-021-02917-1)
Supplement: Supplementary file 14 — Reporting Summary [file 42003_2021_2917_MOESM14_ESM.pdf]

nature research

Corresponding author(s): Shuo Wang

Last updated by author(s): Apr 3, 2021

Reporting Summary

Nature Research wishes to improve the reproducibility of the work that we publish. This form provides structure for consistency and transparency in reporting. For further information on Nature Research policies, see our Editorial Policies and the Editorial Policy Checklist.

Statistics

For all statistical analyses, confirm that the following items are present in the figure legend, table legend, main text, or Methods section.

n/a	Confirmed	
☐	☒	The exact sample size (n) for each experimental group/condition, given as a discrete number and unit of measurement
☐	☒	A statement on whether measurements were taken from distinct samples or whether the same sample was measured repeatedly
☐	☒	The statistical test(s) used AND whether they are one- or two-sided<br>*Only common tests should be described solely by name; describe more complex techniques in the Methods section.*
☐	☒	A description of all covariates tested
☐	☒	A description of any assumptions or corrections, such as tests of normality and adjustment for multiple comparisons
☐	☒	A full description of the statistical parameters including central tendency (e.g. means) or other basic estimates (e.g. regression coefficient) AND variation (e.g. standard deviation) or associated estimates of uncertainty (e.g. confidence intervals)
☐	☒	For null hypothesis testing, the test statistic (e.g. F, t, r) with confidence intervals, effect sizes, degrees of freedom and P value noted<br>*Give P values as exact values whenever suitable.*
☒	☐	For Bayesian analysis, information on the choice of priors and Markov chain Monte Carlo settings
☒	☐	For hierarchical and complex designs, identification of the appropriate level for tests and full reporting of outcomes
☐	☒	Estimates of effect sizes (e.g. Cohen's d, Pearson's r), indicating how they were calculated

Our web collection on statistics for biologists contains articles on many of the points above.

Software and code

Policy information about availability of computer code

Data collection	Matlab, Psychtoolbox, Neuralynx, Eyelink 1000
Data analysis	Custom code written in Matlab; Matlab neural decoding toolbox (NDT); VGG-16 deep neural network (DNN) implemented in Matlab

For manuscripts utilizing custom algorithms or software that are central to the research but not yet described in published literature, software must be made available to editors and reviewers. We strongly encourage code deposition in a community repository (e.g. GitHub). See the Nature Research guidelines for submitting code & software for further information.

Data

Policy information about availability of data

All manuscripts must include a data availability statement. This statement should provide the following information, where applicable:

- Accession codes, unique identifiers, or web links for publicly available datasets
- A list of figures that have associated raw data
- A description of any restrictions on data availability

All data that support the findings and conclusions of this study will be deposited in the OSF repository upon publication of the study.

Field-specific reporting

Please select the one below that is the best fit for your research. If you are not sure, read the appropriate sections before making your selection.

☒ Life sciences ☐ Behavioural & social sciences ☐ Ecological, evolutionary & environmental sciences

For a reference copy of the document with all sections, see nature.com/documents/nr-reporting-summary-flat.pdf

Life sciences study design

All studies must disclose on these points even when the disclosure is negative.

Sample size	Sample size was determined based on our previous publications (Wang et al. PNAS 2014; Wang et al. Nat Commun 2017; Wang et al. Curr Biol 2018; Wang et al., Brain 2019).
Data exclusions	Four sessions of data were excluded because of substantial missing eye movement data. Data exclusion criteria have been described in the manuscript.
Replication	Results were replicated with different datasets, which has been described in the manuscript.
Randomization	Participants were not grouped and hence no randomization was performed. Trial order was fully randomized for each subject.
Blinding	Data collection was performed automatically and experimenters were blind to the experimental conditions (occurence of catch trials). No blinding was involved during data analysis, as we relied on a fully automatized procedure that did no require data manipulation.

Reporting for specific materials, systems and methods

We require information from authors about some types of materials, experimental systems and methods used in many studies. Here, indicate whether each material, system or method listed is relevant to your study. If you are not sure if a list item applies to your research, read the appropriate section before selecting a response.

Materials & experimental systems

n/a	Involved in the study	
☒	☐	Antibodies
☒	☐	Eukaryotic cell lines
☒	☐	Palaeontology and archaeology
☒	☐	Animals and other organisms
☐	☒	Human research participants
☒	☐	Clinical data
☒	☐	Dual use research of concern

Methods

n/a	Involved in the study	
☒	☐	ChIP-seq
☒	☐	Flow cytometry
☒	☐	MRI-based neuroimaging

Human research participants

Policy information about studies involving human research participants

Population characteristics	Five neurosurgical patients with pharmacologically intractable epilepsy participated in the study. Their detailed characteristics have been shown in Supplementary Table 1 in the manuscript.
Recruitment	Participants were recruited through neurosurgery clinic. No systematic recruitment bias were expected to alter the results.
Ethics oversight	This research was approved by the WVU IRB protocol 1709745061: Investigating human cognition with medically necessary intracranial electrophysiologic recordings

Note that full information on the approval of the study protocol must also be provided in the manuscript.
